# What role can decentralized trial designs play to improve rare disease studies?

**DOI:** 10.1186/s13023-022-02388-5

**Published:** 2022-06-20

**Authors:** J. Moore, N. Goodson, P. Wicks, J. Reites

**Affiliations:** 1 THREAD Research, 155 El Camino Real, Tustin, CA 92780 USA; 2 Wicks Digital Health, Advantage House, Lichfield, WS13 6AQ Staffordshire UK

**Keywords:** Decentralized clinical trials, Rare diseases, Telemedicine

## Abstract

People affected by rare diseases want to be involved in research and the search for new treatments. Randomized controlled trials remain the best way of finding new interventions, but many elements of traditional study design are not best suited for rare diseases. Barriers to patients and families include the use of specialist hospital sites for recruitment, requiring frequent site-based study visits for data collection, and a high burden of tests and outcome measures in research. While decentralized clinical trial (DCT) designs have been developed in some rare disease trials, changes necessitated by the COVID-19 pandemic present an opportunity for them to become a standard approach. DCT approaches have been shown to be more resilient to changes in enrolment and attrition during COVID-19 than traditional designs and offer benefits in terms of patient burden, convenience, inclusion, and data quality. Digital tools such as wearable devices and electronic clinical outcome assessments may also provide more convenient and environmentally valid measures of how a condition affects the life of an individual in their regular environment (e.g. mobility around the home versus a hospital corridor). Digital solutions have greater ability to support language localization, accessibility, and may lead to increase access to global rare disease trials. In parallel, challenges exist, such as the technical support, the digital divide, ensuring high quality data, and delivering safe trials.

## Introduction

### Overview

Rare diseases are not all that rare; directly affecting an estimated 3.5–5.9% of the global population, some half a billion people [1]. In recent years, rare disease therapies have accounted for nearly half of new drug approvals [2]. Despite the disease burden and wave of new treatments, most rare disease conditions lack any effective treatment. Rare diseases are associated with a shortened lifespan, high healthcare utilization, significant disability, and a substantial cost of illness [3], necessitating rapid development of novel therapies to improve patient outcomes.

Regulatory agencies have provided novel mechanisms for rare disease approvals, driving efforts from biotechnology companies and ultimately resulting in significant therapeutic pipelines. However, there remain challenges associated with clinical trials in rare diseases, including the geographic dispersion of patients, a lengthy diagnostic process, more complex study protocols and endpoints, a short prognosis with brief window in which to intervene, and a compounding effect of intersectional socioeconomic burdens placed on the family [4]. When participants, facing this uphill battle, are additionally required to travel long distances for lengthy site visits, it can significantly strain their ability to fully engage in research activities [5]. These issues are compounded for members of historically marginalized communities who may face additional intersectional barriers [6]. Despite many obstacles, rare disease patients have consistently demonstrated willingness to overcome hurdles to participate in research [7], but the structure and approach of traditional clinical trials leaves many excluded.

### Impact of COVID on rare trials

The COVID-19 pandemic has added an extra layer of complexity to pre-existing operational challenges such as the requirement for patients with rare diseases to shield themselves at home, manage disruptions to their existing medications, and even access basic necessities [8]. In response, regulators such as the US Food and Drug Administration (FDA) have suggested “trials that incorporate components of decentralization, digital health, electronic platforms, and other technologies may ease the burden of participating in a clinical trial.” [9] While the past 15 years has seen an expansion in the rare field’s use of innovative technologies [10] such as wearable devices [11], electronic clinical outcome assessment (eCOA), and electronic patient reported outcomes (ePROs) [12], these have tended to focus on exploratory endpoints, recruitment, and observational registries rather than taking a central role in trial design. With experts predicting the transition of COVID-19 from pandemic to endemic, now is an excellent time to consider normalizing decentralized approaches in rare disease [13].

Even before the pandemic, institutions were testing the validity and quality of care when delivered via telemedicine, home health care, remote sensors, and mobile device applications in rare disease populations [14]. As patients and medical care providers integrate technological solutions into the traditional medical model, it is only natural that clinical research incorporates similar methodologies into trial design.

This change in care paradigm has led several rare disease organizations to embrace decentralized approaches. For example, the European Cystic Fibrosis Society-Clinical Trials Network (ECFS-CTN) recently proposed that the use of home assessments, video and phone calls, electronic consent, and home delivery of study drugs might mitigate the impact of disruption wrought by COVID-19 [15]. The Parent Project Muscular Dystrophy (PPMD) Duchenne Registry moved to a decentralized mobile app allowing eConsent, eCOAs, online surveys, and other decentralized tools, yielding a 50% increase in survey data collected during 2020 compared to the previous year [16]. More broadly, patient organizations who adopted digital communication systems to maintain contact with their network were better able to maintain their operations [17]. The goal of these decentralized approaches is not to remove hands-on interactions between clinicians and patients, but rather to integrate technology in a way that collects reliable data at decreased burden for the rare-disease community.

## Application of decentralized methods in rare disease studies

Decentralized methods transfer assessments that previously occurred in centralized medical facilities to other locations such as the participant’s home, local clinics, or digital interactions through applications on a mobile device or computer. Rare diseases require specific considerations regarding the needs of the population both for clinical care and study design. However, based on our experience of over 50 trials in rare conditions with traditional and decentralized study designs including amyotrophic lateral sclerosis (ALS), spinal muscular atrophy (SMA), muscular dystrophy (MD), and Alpha-1 antitrypsin deficiency (AATD), DCT design elements can be valuable across study designs and phases (see Table 1). Certain sub-populations of the rare disease community merit special considerations; those affected with rare oncology indications, pediatric rare diseases, and the ultra-rare disease.

**Table 1:** Optimal application of decentralized trial design elements across study phases (+)

Phase	Online Recruitment	Electronic Consent (Remote eConsent)	Electronic Consent (On-Site eConsent)	Electronic Patient Education + Engagement	Electronic Clinical Outcome Assessments (eCOAs)	Telehealth	Wearable Sensors	Home Health Visits by Healthcare Professionals	Drug Supply by Mail
Natural History Study/Registry	+	+		+	+	+	+		
First in Human/Ph1			+	+	+	+			
Phase II/III	+		+	+	+	+	+	+	+
Open Label Extension		+		+	+	+	+	+	+
Post-Approval Observational	+	+		+	+	+	+		
Long-Term Followup		+		+	+	+	+		

### DCT considerations for special populations: rare oncology

Trials for rare oncology conditions often include genetic testing, radiotherapy, advanced imaging, and methods of therapy delivery not suitable for a home environment. Despite these limitations, the FDA’s Oncology Center of Excellence have requested that in new submissions sponsors voluntarily flag data to discriminate between assessments gathered "remotely” and “trial sites.” The hope is that knowledge gained from these datasets will highlight opportunities and challenges with decentralized approaches [18]. While still emerging, some published evidence suggests there are robust mechanisms to incorporate DCT elements, for example telehealth virtual visits, eCOA, and ePRO in oncology rare disease studies [19–22].

### DCT considerations for special populations: pediatric rare diseases

Families affected by pediatric rare diseases may be more likely than other trial populations to be affected by caregiving responsibilities for other children, who may in turn themselves be affected with the same condition as their participating sibling. Because pediatric genetic diseases are more likely to be fatal before the age of five (5) [3], the caregivers of young children may also have work responsibilities, which can bear an economic toll of participation in traditional site-based visits. DCT approaches that offer telehealth and at-home visits with medication delivered by post or administered by a healthcare professional closer to home will have significant benefits in reducing burden for patients and their families. The use of wearable devices shows significant opportunity in ecologically valid continuous data sampling for movement, which has been shown to correlate well with standardized measures such as the six-minute walk test [23]. Additional benefits include giving families insight into how well their child may be progressing and increasing engagement in data collection.

### DCT considerations for special populations: ultra-rare disease

There are inconsistently defined subgroups of rarity within the rare disease space [1]. For example, it is estimated that some 80% of “rare patients” have just 4.2% of the “rare diseases” whereas 85% of “rare diseases” are *extremely* rare, with a prevalence below one (1) person per million. If we were to consider designing a study that might affect a dozen or so individuals in a country like the UK or Germany, and perhaps a hundred or so across the entire United States, a traditional site-based approach is likely to be relatively slow to recruit and costly to operationalize. It is not unheard of for ultra-rare patients to be flown across country or even internationally to take part in studies, a hugely disruptive and costly experience. Identifying and contracting with relevant sites can be a substantial barrier to trial startup and the need to train and qualify an array of site personnel to administer tests, interviews, and questionnaires for what will be a very small sample is inefficient. DCT features such as telehealth, eCOA, ePROs, eDiaries, image capture, voice capture, and wearables have the potential to produce more consistent data by reducing rater bias and the ability to deploy patient smartphone applications in multiple languages could make the user experience more consistent while keeping participants connected to the very few global care teams who may be able to effectively treat them.

### Challenges for decentralized approaches in rare disease

Limitations still to be fully addressed include harmonizing data across geographies, regulatory gaps (e.g. remote eConsent or electronic signature may not be allowed in certain countries or regions), varying regulatory expectations, health technology assessment and payer evidence needs, and vigilance with regards to data quality [15]. Where sponsors or study designers may have concerns about the implementation of technology in rare disease, there are data to support their use [24, 25] but not in every disease. Where peer-reviewed data is lacking, we have found that consulting directly with patients, caregivers, and clinicians reveals numerous modifications in care and research that were adapted dynamically throughout the COVID-19 pandemic [8].

## Conclusion

By the end of 2019, the biomedical research enterprise has developed 564 orphan products to treat 838 rare diseases with a traditional and mostly site-based approach [26]. But for all the lives changed, this still represents less than 5% of total rare diseases globally [27]; patients need effective treatments faster. During the last two decades, consumer technology like smartphones and social media has allowed patient communities to connect, raise awareness, share experiences, and advance treatment practices across borders in a massively decentralized fashion [28]. While the COVID-19 pandemic has had a negative impact on all those living with rare diseases [8], if there are any silver linings, one might be the rapid demonstration that remote and decentralized technologies can be introduced, adapted, and tailored to the care and research environment to help those living and working with rare disease.

## Data Availability

Not applicable.
